# Three-year Outcomes After Conversion From Monthly to Every 2-month Belatacept Maintenance Therapy in Kidney Transplant Recipients: Results From a Randomized Controlled Trial

**DOI:** 10.1097/TXD.0000000000001449

**Published:** 2023-02-08

**Authors:** Aileen C. Johnson, Geeta M. Karadkhele, Neeta Shenvi, Kirk A. Easley, Christian P. Larsen, I. Raul Badell

**Affiliations:** 1 Emory Transplant Center, Atlanta, GA.; 2 Department of Biostatistics and Bioinformatics, Rollins School of Public Health, Emory University, Atlanta, GA.

## Abstract

Maintenance immunosuppression with belatacept following kidney transplantation results in improved long-term graft function as compared with calcineurin inhibitors. However, broad application of belatacept has been limited, in part related to logistical barriers surrounding a monthly (q1m) infusion requirement.

**Methods.:**

To determine whether every 2-mo (q2m) belatacept is noninferior to standard q1m maintenance, we conducted a prospective, single-center randomized trial in low-immunologic-risk, stable renal transplant recipients. Here, post hoc analysis of 3-y outcomes, including renal function and adverse events, are reported.

**Results.:**

One hundred sixty-three patients received treatment in the q1m control group (n = 82) or q2m study group (n = 81). Renal allograft function as measured by baseline-adjusted estimated glomerular filtration rate was not significantly different between groups (time-averaged mean difference of 0.2 mL/min/1.73 m^2^; 95% confidence interval: −2.5, 2.9). There were no statistically significant differences in time to death or graft loss, freedom from rejection, or freedom from donor-specific antibodies (DSAs). During the extended 12- to 36-mo follow-up, 3 deaths, 1 graft loss occurred in the q1m group, compared with 2 deaths, and 2 graft losses in the q2m group. In the q1m group, 1 patient developed DSAs and acute rejection. In the q2m group, 3 patients developed DSAs and 2 associated with acute rejection.

**Conclusions.:**

Based on the similar renal function and survival at 36 mo compared with q1m, q2m belatacept is a potentially viable maintenance immunosuppressive strategy in low immunologic risk kidney transplant recipients that may facilitate increased clinical utilization of costimulation blockade-based immunosuppression.

Kidney transplant is the treatment of choice for end-stage renal disease and has proven benefits on both life expectancy and quality of life.^1,2^ Research and advancements in clinical care have improved short-term outcomes for renal transplant recipients to >90% graft survival at 1-y posttransplant.^3^ However, long-term outcomes have remained relatively stable for decades with 10-y graft survival ranging from 50% to 70%.^4,5^ Although the etiology of late graft loss is not completely understood, leading factors include immunosuppression-related nephrotoxicity and chronic immunologic allograft injury associated with alloantibody-mediated rejection.^6,7^ Belatacept was developed as an alternative to the nephrotoxic calcineurin inhibitors (CNIs) used most frequently as maintenance immunosuppression, and has demonstrated improvements in patient and graft survival, renal function, and the incidence of donor-specific antibodies (DSAs) over CNI therapy in long-term follow-up.^8-10^

Despite superior long-term outcomes on belatacept therapy, several barriers have prevented large-scale adoption as rejection prophylaxis in kidney transplantation, with >90% of new renal transplant recipients still initiated on CNI-based maintenance immunosuppression.^3^ Although initial concerns were related to higher acute cellular rejection (ACR) rates on belatacept,^11,12^ multiple subsequent regimens have achieved acceptable ACR rates, comparable to that of tacrolimus-based therapy.^13-15^ With improved management of this biologic hurdle, additional barriers to belatacept adoption are largely logistic and relate to need for intravenous (IV) access and infusion administration.^16^ In its current form, belatacept requires IV infusions on a monthly basis. The need for consistent vascular access is not always trivial in this end-stage renal disease population nor is the requirement for monthly visits to an infusion center, which often incurs costs that compound the expense of infusions alone. In addition, the transplant center is challenged to acquire and maintain staffing for an infusion center, as well as to coordinate with external infusion centers for recipients outside the local area.

Although these burdens may be worth the benefit accrued by patients in avoiding nephrotoxic medications and decreasing their risk of DSAs and antibody-mediated rejection (AMR),^9,10^ overcoming these barriers is of great interest and will facilitate belatacept uptake and improve outcomes. Early phase II investigations suggested that dosing belatacept every 8 (q8) wk, in contrast to standard of care 4-wk (q4) dosing, may provide sufficient immunosuppression.^17,18^ Analysis of q4 and q8 week dosing of belatacept in the phase II clinical trial demonstrated improved long-term graft function in belatacept patients compared with cyclosporine. However, the study was not powered to detect differences between q4 and q8 week groups, confounding interpretation of the safety of alternative dosing strategies. Nonetheless, because the highest density of acute rejection occurred in the first year posttransplant, the favorable long-term outcomes prompted consideration of a transition from monthly to every 2-mo (q2m) dosing in low immunological risk patients greater than 1 y posttransplant.

We conducted a randomized controlled trial designed to test this possibility that demonstrated noninferior renal function with q2 month dosing at 1 y.^19^ Although acute rejection episodes were low and heavily related to medication nonadherence, there was a trend toward better rejection-free survival in the q1m group. Here, we present the 3-y follow-up of these patients, including renal function, immunologic outcomes, and adverse events.

## MATERIALS AND METHODS

### Randomized, Noninferiority Trial Design

Between October 2015 and August 2019, eligible patients were enrolled as previously described into a 12-mo, randomized, parallel-group, single-center study.^19^ Low-immunological-risk adult kidney transplant recipients at least 1-y posttransplant were considered for inclusion. Study participants were required to have completed transient CNI therapy a minimum of 6 mo before enrollment. Additional inclusion criteria were stable estimated glomerular filtration rate (eGFR) >35 mL/min/1.73 m^2^, and minimum maintenance immunosuppression of belatacept (5 mg/kg monthly), mycophenolate mofetil (1000 mg daily), and prednisone (5 mg daily). Patients were excluded for high immunological risk if they had calculated panel reactive antibodies >50, positive DSA, or >1 prior rejection episode. Additional exclusion criteria included presence of a nonrenal solid organ transplant, uncontrolled diabetes (Hgb A1c > 8%), proteinuria, and active infection or viremia.

### Three-y Follow-up

The outcomes of all enrolled patients were analyzed retrospectively out to 3 y of follow-up, as approved by the Emory Institutional Review Board (STUDY00003425). At the end of the 12-mo protocol study period, patients allocated to the q1m reference group were maintained on standard monthly belatacept (5 mg/kg) infusions, and those allocated to the q2m group were maintained on bimonthly infusions at the same dose. Infusions were administered at the Emory Transplant Center or a certified local infusion center. Patients were monitored per standard of care for years 2–3 of follow-up. Patients experiencing acute rejection were treated with established rejection grade-based protocols (corticosteroids for grades <1B and thymoglobulin for grades ≥1B), and q2m participants were converted back to monthly belatacept dosing. Subsequent to the initial 12-mo study period, there was no further protocol HLA antibody evaluation. Patients were evaluated for changes in clinical management related to CMV and BK viremia. Patients enrolled in the initial randomized controlled trial who died or withdrew before beginning treatment were excluded from all analyses (including the original protocol analysis).

### Outcomes and Analyses

The primary objective of this follow-up study was to assess whether q2m belatacept dosing continues to perform similarly to standard monthly maintenance therapy as measured by renal function (eGFR) throughout the 36 mo following randomization. Renal function was calculated from serum creatinine using the CKD-EPI equation. Secondary outcome measures included assessment of rejection, DSAs, graft loss, and death. Biopsies were performed for cause, with rejection defined as ≥grade 1A according to standard Banff criteria as determined by a staff pathologist.^20^ Data analyses were performed according to subjects’ original treatment assignment regardless of compliance (ie, intent-to-treat [ITT]). All statistical tests were 2-sided, and a *P* value ≤0.05 was considered statistically significant.

The primary endpoint in the original ITT, parallel-group noninferiority trial was mean eGFR from baseline to 12 mo, using a noninferiority margin of 6.0 mL/min/1.73 m^2^. Here, we analyzed 36-mo eGFR outcome data in a secondary analysis. To account for uneven sampling, months 12–36 were divided into 3-mo intervals, and the average value of eGFR for each patient over the interval was calculated. A repeated-measures analysis of eGFR was performed with a means model via the SAS MIXED Procedure (version 15.1; SAS Institute, Cary, NC), providing separate estimates of the mean eGFRs by time-on-study and treatment group. A first-order autoregressive variance-covariance form in repeated measurements was found to be optimal for eGFR and robust estimates of the standard errors of parameters were used to perform statistical tests and construct 95% confidence intervals (CIs).^21,22^ The statistical test for interaction between time-on-study and treatment was the overall hypothesis test to determine whether eGFR in the 2 study groups changed in significantly different ways during the follow-up period.

Since the mean eGFR in the 2 treatment groups was consistently similar over time, the time-averaged mean difference in eGFR and its 95% CI between subjects randomized to q1m or randomized to q2m was reported. A baseline-adjusted analysis was also performed for eGFR. Two additional sensitivity analyses were conducted, each using baseline-adjusted eGFR. The first sensitivity analysis was comparison of time-averaged eGFR conditional on patient and graft survival. The second sensitivity analysis included all patients with imputation of zeros for patients who experienced death or graft loss at all time points subsequent to the event.

The secondary outcomes of rejection, DSAs, death, and graft loss were analyzed ITT including all randomized subjects who received treatment using Cox regression and Kaplan–Meier time-to-event comparisons between groups and the log-rank test to determine statistical significance. For Kaplan–Meier analysis, patients were censored when lost to follow-up (q1m = 4, q2m = 1), all of which were related to relocation during the study period. For survival analysis directed toward immune endpoints, patients who died during the study period were excluded as this event represented a population with nonarbitrary risk related to the overall study population. Analysis of adverse events was performed using R, version 4.0.2.

## RESULTS

### Patient Disposition

**FIGURE 1.**
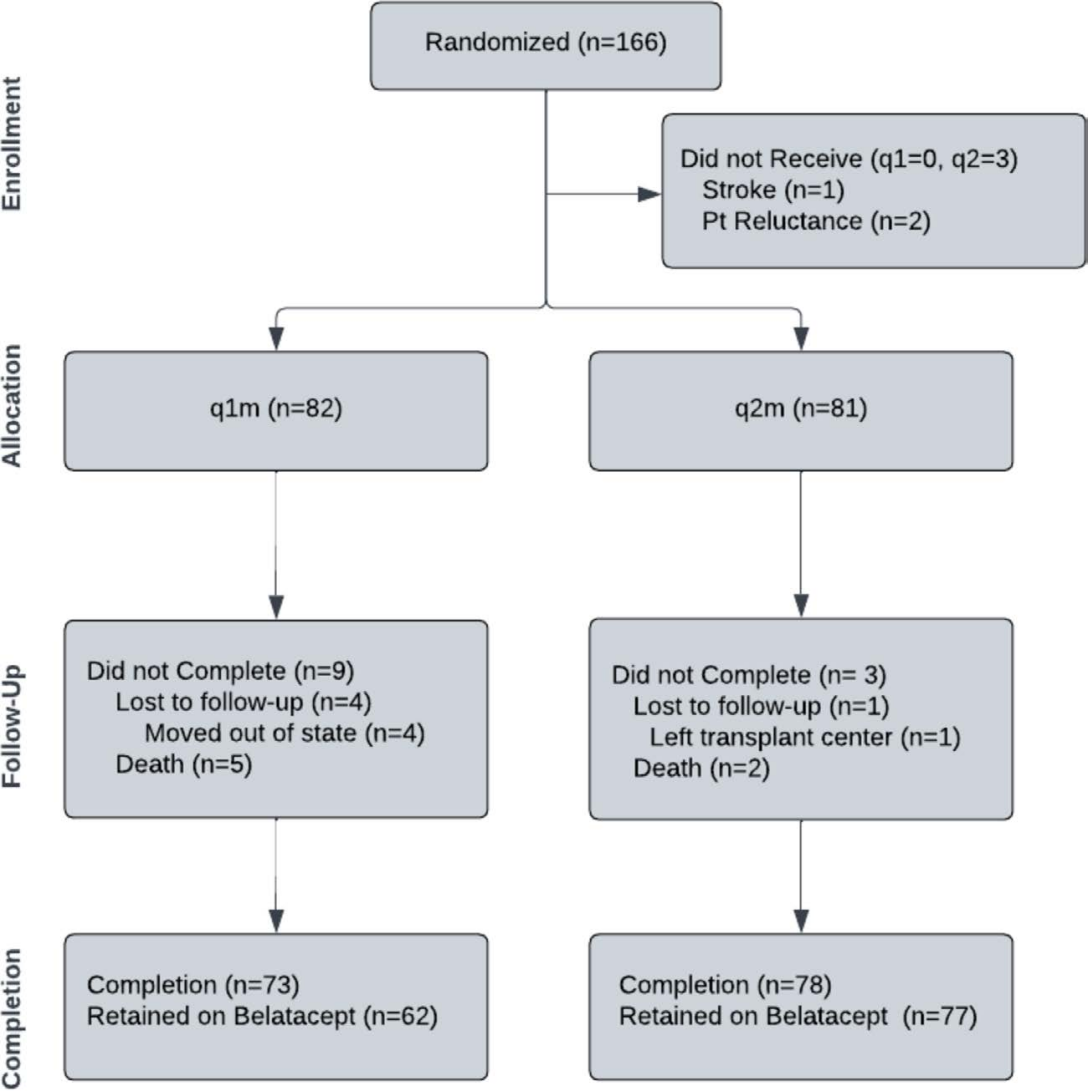
Patient enrollment diagram. After initial evaluation for the 12-mo trial, patients were randomized to monthly or every 2-mo belatacept therapy. Retrospective analysis of 3-y outcomes was performed for all patients who received therapy after randomization. q1m, every mo dosing; q2m, every 2-mo dosing of belatacept.

Randomized treatment groups were balanced in the distribution of demographic, clinical‚ and immunological risk factors, including times posttransplant and off CNI (Table 1). Overall, 151 of 163 (93%) subjects achieved 3 y of follow-up at our center, representing 73 of 82 (89%) subjects in the q1m group and 78 of 81 (96%) in the q2m group (Figure 1). Four subjects were lost to follow-up in the q1m group, all of whom relocated out of state during the study. The one subject lost to follow-up in the q2m group transferred to a different transplant center. In the q1m group, there were a total of 5 deaths, 2 in the first year and 3 during the second and third years of follow-up. In the q2m group, 2 subjects died, both during the third year of follow-up.

**TABLE 1.** Patient characteristics

	q1m (n = 82)	q2m (n = 81)
Age, y (SD)	52 (12)	50 (13)
Sex		
Male	63 (77)	54 (67)
Female	19 (23)	27 (33)
Race		
Black	32 (39)	36 (44)
Non-Black	50 (61)	45 (56)
Etiology of ESRD		
Hypertension	19 (23)	22 (27)
Diabetes	17 (21)	18 (22)
PKD	14 (17)	11 (14)
Glomerulonephritis	7 (9)	2 (3)
FSGS	5 (6)	6 (7)
Other	20 (24)	22 (27)
Donor type		
Living	46 (56)	37 (46)
Deceased	36 (44)	44 (54)
Time posttransplant, mo (IQR)	26 (20–47)	22 (19–34)
CMV risk		
Low	20 (24)	18 (22)
Moderate	56 (68)	54 (67)
High	6 (7)	9 (11)
cPRAs at transplant		
0	71 (87)	67 (83)
<20	6 (7)	6 (7)
≥20	5 (6)	8 (10)
Induction immunosuppression		
Thymoglobulin	1 (1)	3 (4)
Basiliximab	81 (99)	78 (96)
Maintenance immunosuppression		
Belatacept 1.0	12 (15)	7 (9)
Belatacept 2.0	70 (85)	74 (91)
Time off CNIs, d (IQR)	393 (237–592)	332 (245–700)
eGFR, mL/min/1.73 m^2^ (SD)	72.4 (17.7)	69.3 (16.4)
Biopsy history		
0	43 (52)	33 (41)
≥1	39 (48)	48 (59)
Borderline	8 (10)	15 (19)
Rejection history		
Total	8 (10)	12 (15)
IA, IB	4 (5)	7 (9)
IIA	4 (5)	5 (6)

Data are mean (SD), number (%), or median (IQR).

Belatacept 1.0, belatacept-based CNI-free regimen; Belatacept 2.0, belatacept-based regimen with transient CNI therapy; CMV, cytomegalovirus; CNI, calcineurin inhibitors; cPRA, calculated panel reactive antibody; DSA, donor-specific antibody; eGFR, estimated glomerular filtration rate; ESRD, end-stage renal disease; FSGS, focal segmental glomerulosclerosis; IQR, interquartile range; PKD, polycystic kidney disease; q1m, every mo dosing; q2m, every 2-mo dosing of belatacept.

Of patients on a q1m regimen, 11 transitioned off belatacept therapy during the study period. The most common reason for this transition was cost (n = 5), followed by side effects (n = 3), and infectious complications (n = 1). In contrast, only 1 patient in the q2m group transitioned off belatacept therapy, secondary to recurrence of monoclonal gammopathy of undetermined significance. One patient from the q1m group was decreased to a q2m dosing frequency after developing infectious complications. Eight patients enrolled in q2m belatacept dosing ended the study period on standard belatacept dosing (q1m), most commonly related to the development of DSAs or rejection (n = 5), followed by patient preference (n = 3). In total, of living subjects with a functioning graft, 86% of q1m (62 of 72) and 99% of q2m (75 of 76) remained on belatacept at 3-y follow-up.

### Renal Function

**FIGURE 2.**
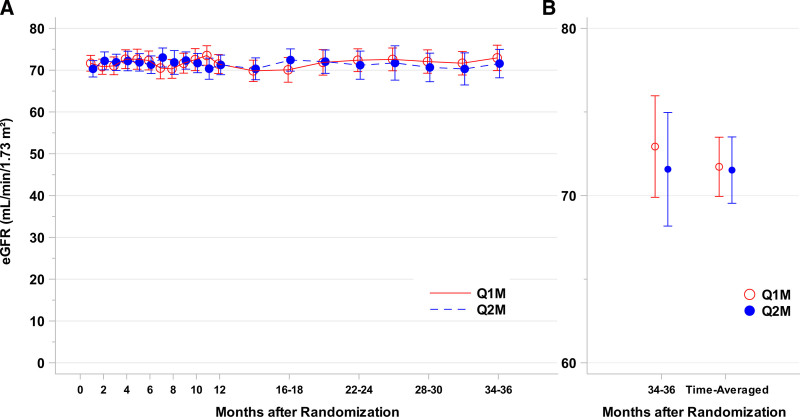
Renal function on q2m belatacept therapy is equal to standard q1m therapy over 3-y follow-up. Longitudinal baseline-adjusted eGFR data for q1m (red) and q2m (blue) are shown in (A). There was no statistically significant difference in eGFR between groups at any time point. B, 36-mo time point and time-averaged mean for q1m and q2m from the baseline-adjusted model. eGFR, estimated glomerular filtration rate; Q1m, every mo dosing; Q2m, every 2-mo dosing of belatacept.

Renal function as measured by eGFR was the same between q1m and q2m groups 3 y after randomization. There was no statistically significant difference in time-averaged eGFR nor eGFR at 36 mo (Figure 2A, B). Table 2 outlines eGFR analysis between q1m and q2m belatacept dosing regimens. Time-averaged eGFR unadjusted for baseline was 72.9 mL/min [69.1, 76.8] in the q1m group compared with 69.9 [66.2, 73.5] in the q2m group (*P* = 0.26). The mean difference between groups (q1m − q2m) was 3.1 [−2.3, 8.4]. After adjustment for baseline eGFR (Figure 2A), the difference between groups reduced further with a mean of 71.7 [70.0, 73.5] in the q1m group compared with 71.5 [69.5, 73.5] in the q2m group (*P* = 0.88). This resulted in a mean difference between groups of 0.2 [−2.5, 2.9] (Figure 2B). Similar to the time-averaged means, the 36-mo time point in the adjusted model also showed no difference between q1m and q2m groups (q1m: 72.9 [69.9, 76.0], q2m: 71.6 [68.2, 75.0]). Number-at-risk and unadjusted/adjusted mean eGFR with 95% CIs for each time point are available in **Tables S1 and S2, SDC**, http://links.lww.com/TXD/A505.

**TABLE 2.** Time-averaged mean eGFR (mL/min/1.73 m^2^) during 36 mo of follow-up by monthly or every 2-mo belatacept maintenance therapy

	Measurements	Patients	Mean eGFR	95% CI	Mean difference	95% CI	*P*
Adjusted					0.2	−2.5, 2.9	0.88
Q1m	1439	82	71.7	70.0, 73.5			
Q2m	1515	81	71.5	69.5, 73.5			
Conditional					0.1	−2.0, 2.2	0.91
Q1m	1372	76	72.5	71.0, 74.0			
Q2m	1442	77	72.4	70.9, 73.9			
Imputed					−1.3	−4.6, 2.0	0.43
Q1m	1492	82	69.8	67.2, 72.4			
Q2m	1522	81	71.1	69.1, 73.1			
Unadjusted					3.1	−2.3, 8.4	0.26
Q1m	1521	82	72.9	69.1, 76.8			
Q2m	1596	81	69.9	66.2, 73.5			

CI, confidence interval; eGFR, estimated glomerular filtration rate; Q1m, every mo dosing; Q2m, every 2-mo dosing of belatacept.

Sensitivity analyses confirmed these results. In the conditional model (sensitivity analysis 1), excluding patients who experienced death or graft loss, the mean eGFR in the q1m group was 72.5 [71.0, 74.0] versus 72.4 [70.9, 73.9] in the q2m group (*P* = 0.91, Table 2). The mean difference between groups in the conditional model was 0.1 [−2.0, 2.2]. When eGFR was imputed as zero subsequent to the event for patients who experienced death or graft loss (imputed model, sensitivity analysis 2), the mean for q1m patients was 69.8 [67.2, 72.4] as compared with 71.1 [69.1, 73.1] for q2m patients (*P* = 0.43, Table 2). The mean difference between groups in the imputation model was −1.3 [−4.6, 2.0]. Number-at-risk and mean eGFR with 95% CIs for each time point for these sensitivity analyses are available in **Tables S3 and S4, SDC**, http://links.lww.com/TXD/A505.

### Patient and Graft Survival

**FIGURE 3.**
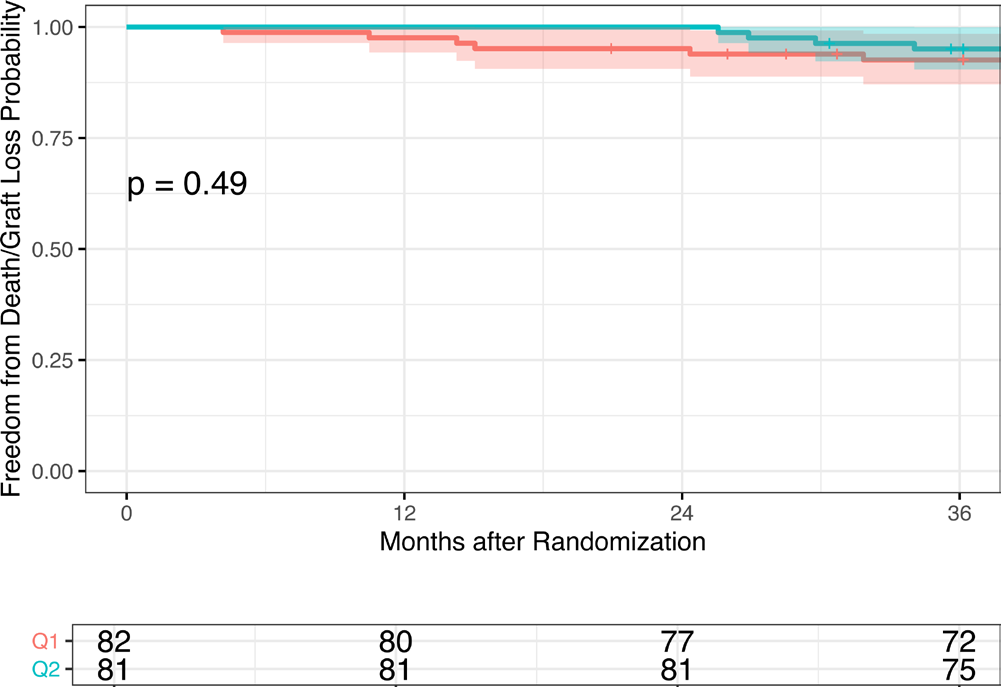
Kaplan–Meier time to event curve for patient death/graft loss. Kaplan–Meier plot for freedom from combined death and graft loss.

Throughout the initial 12-mo study period, there were no graft losses in either group and 2 deaths in the q1m group. During years 2–3 of follow-up, there were 3 deaths, 1 graft loss in the q1m group and 2 deaths, 2 graft losses in the q2m group. We observed no significant differences in the rate of the combined outcome of death or graft loss in patients who received q2m compared with q1m belatacept (hazard ratio, 0.64 [95% CI, 0.18, 2.27]) nor in survival to the combined outcome as examined by the Kaplan–Meier analysis (*P* = 0.49, Figure 3). The 36-mo cumulative survival was 92.6% [87.1, 98.5] in the q1m cohort and 95.0% [90.4, 99.9] in the q2m cohort (**Table S5, SDC**, http://links.lww.com/TXD/A505).

The 3 additional deaths in the q1m group were patients 009, 024, and 153. Patient 009 died at home on study day 736, patient 153 on study day 939, both from unknown causes and with a functioning graft. Patient 024 died secondary to infectious complications related to resistant CMV viremia on study day 429. The 2 deaths in the q2m group were patient 053 who died on study day 890 related to a stroke and patient 147 who died on study day 818 after a myocardial infarction, both with functioning grafts (Table 3). The 3 patients with graft failures independent of death were patient 045 in the q1m cohort and patients 036 and 098 in the q2m cohort. All 3 patients had documented medication nonadherence leading to the development of acute and chronic rejection and subsequent graft failure. Patient 045 had been converted to tacrolimus due to nonadherence with belatacept infusions but remained noncompliant and subsequently developed severe rejection that progressed to graft failure.

**TABLE 3.** Summary of deaths occurring during the study period

Patient ID	Cause of death	Date of death (study d)	Last eGFR before death
Q1m			
112	GNR bacteremia	127	33.09
133	Cryptococcal meningitis	318	83.36
024	CMV viremia	429	13.73
009	Unknown cause	736	48.75
153	Unknown cause	939	52.57
Q2m			
147	Stroke	818	80.21
053	Myocardial infarction	890	46.76

CMV, cytomegalovirus; eGFR, estimated glomerular filtration rate; GNR, Gram negative rod; Q1m, every mo dosing; Q2m, every 2-mo dosing of belatacept.

### Immune Events: Rejection and DSAs

During the extended 12- to 36-mo study period, 1 patient in the q1m arm, patient 045, developed acute rejection and DSAs. Patient 045 presented with mixed ACR/AMR, before progressing to graft failure as described above (Table 4). In the q2m group, 3 patients developed DSAs, 2 of which were associated with acute rejection (patients 036 and 049, Table 4). Patients 036 and 049 each had evidence of both ACR and AMR on biopsy. Patient 111 was biopsied for development of new DSAs associated with an elevated creatinine, found to have borderline rejection and treated with a pulse of oral steroids with rapid recovery of eGFR and reduction in DSAs (mean fluorescence intensity [MFI] 3759 from 6847). All rejection and DSA events were associated with documented nonadherence with immunosuppressive medication. Patients 98 and 135 both had episodes of ACR and AMR associated with nonadherence during the first year of the study and subsequent follow-up biopsies for deteriorating renal function during years 1–3 demonstrated recurrent borderline rejections. Follow-up on all immune events occurring in the first year are detailed in **Table S6, SDC**, http://links.lww.com/TXD/A505.

**TABLE 4.** New immunological events during years 2 and 3 of follow-up

ID	Time (M)	Pathology	DSA specificity (max MFI)	Treatment and 3-y outcome
Q1m				
045	13	1B ACR/AMR	A (7359),B (9266),DR (15,712),DQ (14,694)	Thymoglobulin, plasmapheresis/IVIG.Progressed to graft failure.
Q2m				
036	24	1B ACR/AMR	DP (9890),DQ (6360)	Thymoglobulin, plasmapheresis, IVIG.Converted to q1m.Progressed to graft failure.
049	34	1B ACR/AMR	DQ (44,387)	Thymoglobulin, IV steroids, plasmapheresis, IVIG.Converted to q1m.
111	25	Borderline	A (6847)	Oral steroid pulse.Remained on q2m.Last MFI 3759

ACR, acute cellular rejection; AMR, antibody-mediated rejection; DSA, donor-specific antibody; IV, intravenous; MFI, median fluorescence intensity; Q1m, every mo dosing; Q2m, every 2-mo dosing of belatacept.

**FIGURE 4.**
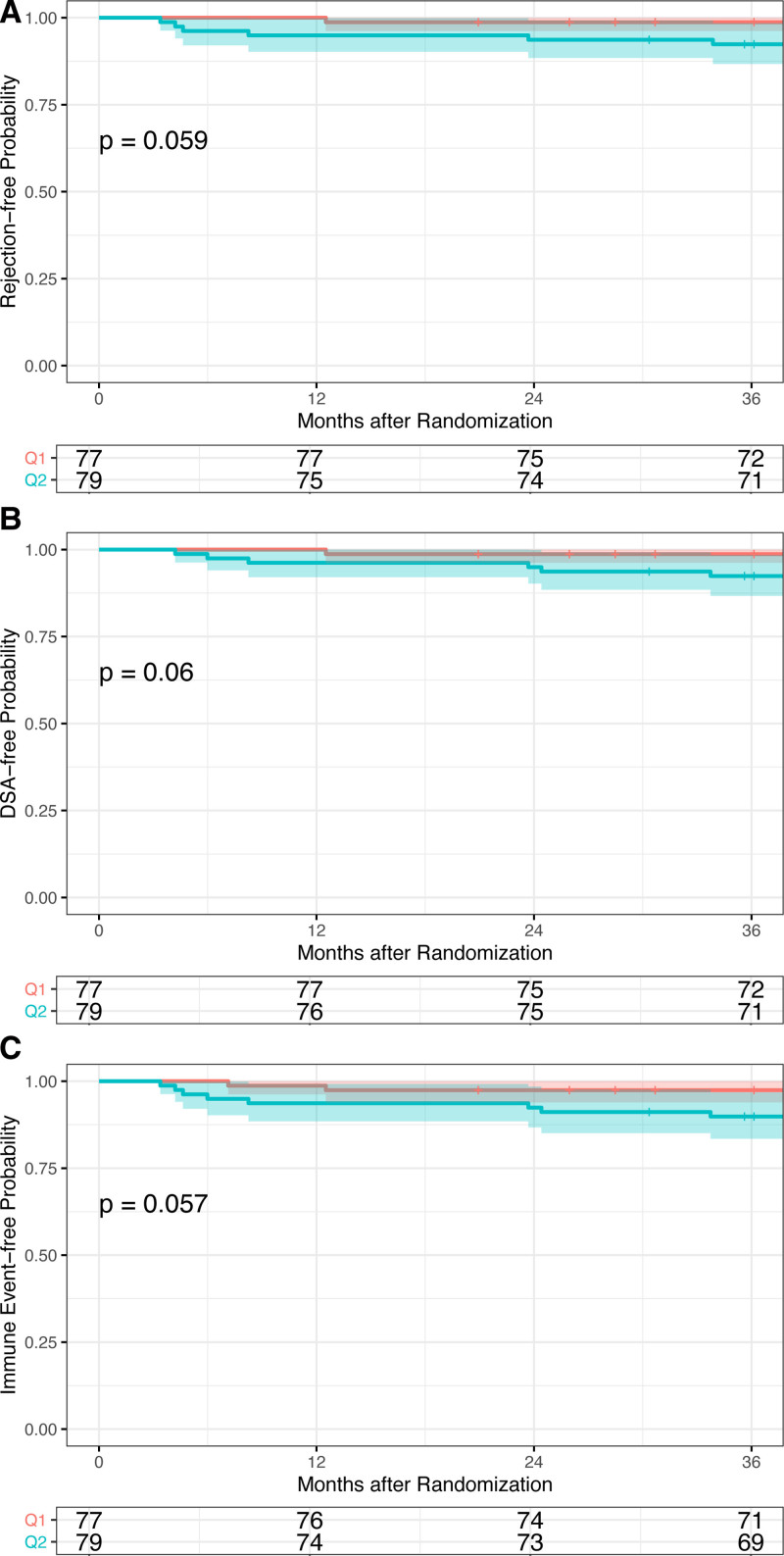
Kaplan–Meier time to event curves for immune events. Kaplan–Meier plots for freedom from (A) rejection, (B) DSAs, and (C) any immune event. DSA, donor-specific antibody.

The Kaplan–Meier analysis of survival to the endpoints of rejection and DSAs demonstrated no significant differences between groups (Figure 4). The cumulative rejection-free survival at 36 mo was 98.7% [96.2, 100] in the q1m cohort and 92.4% [86.7, 98.4] in the q2m cohort (Figure 4, *P* = 0.06; **Table S5, SDC**, http://links.lww.com/TXD/A505). The rate of rejection was not different in patients who received q2m compared with q1m (hazard ratio, 5.99 [95% CI, 0.72, 49.75]). Cumulative DSA-free survival at 36 mo was 98.7% [96.2, 100] in the q1m cohort and 92.4% [86.7, 98.4] in the q2m cohort (Figure 4B, *P* = 0.06). The rate of DSAs was not different in patients who received q2m compared with q1m (hazard ratio, 5.95 [95% CI, 0.72, 49.45]). When viewed as a combined endpoint, cumulative survival free from immune events (rejection ≥ borderline or new DSAs) was not significantly different between groups (q1m: 97.4% [93.9, 100]; q2m: 89.9% [83.4, 96.8]; Figure 4C, *P* = 0.06). Similarly, the rate of combined events was not different in patients who received q2m compared with q1m (hazard ratio, 4.02 [95% CI, 0.85, 18.94]). Additionally, the composite endpoint of rejection, DSAs, death, or graft loss was not different between groups as analyzed by the Kaplan–Meier (*P* = 0.29, **Figure S1, SDC**, http://links.lww.com/TXD/A505).

### Infectious Complications

Four patients in the q1m group (patients 010, 051, 090, 160) and 2 in the q2m group (patients 006 and 073) experienced BK viremia with >10 000 copies (log_10_ > 4) during months 12–36 of the study. Three of 4 patients in the q1m group improved with reduction of immunosuppression. One patient (patient 010) was biopsied in the setting of an elevated serum creatinine, and pathology demonstrated severe BK nephropathy. Despite decreasing the dosing frequency of belatacept, this patient was persistently viremic and was transitioned to tacrolimus-based therapy. Of the 2 patients in the q2m group, one improved with reduction of immunosuppression. The other (patient 006) was biopsied for elevated serum creatinine, received a steroid pulse for BK-related inflammation, and improved with further reduction of mycophenolate mofetil (MMF) (Table 5).

**TABLE 5.** BK virus-related events

Subject	Management	Max	Max (Log_10_)	Biopsy
Q1M				
010	Biopsy for elevated SCr demonstrated severe BVN. Spaced bela to q6, stopped MMF, but remained log_10_ of 6 Transitioned to tacrolimus-based therapy	5e6	6.7	Y
051	Improved with reduced dose MMF	2e5	5.3	N
090	Improved with holding MMF	1e4	4.0	N
160	Improved with reduced dose MMF	1e4	4.0	N
Q2M				
006	Biopsy for elevated SCr demonstrated BK-related inflammation. Improved after steroid pulse and reduction of MMF	1e5	5.0	Y
073	Improved with reduced dose MMF	1e4	4.0	N

BVN, BK virus nephropathy; MMF, mycophenolate mofetil; Q1m, every mo dosing; Q2m, every 2-mo dosing; q6, every 6 wk dosing; SCr, serum creatinine.

Among the overall cohort, only 1 subject, in the q1m group, experienced clinically significant CMV viremia during the study period. Patient 024 developed ganciclovir-resistant CMV that in the context of severe comorbidities and a number of additional infectious complications ultimately led to death.

## DISCUSSION

In this study, we demonstrate that after 3 y, outcomes on q2m belatacept are similar to outcomes on a standard monthly dosing regimen. We detected no difference in time-averaged eGFR between q1m and q2m groups with equivalent renal function at 36 mo. During years 2–3 of follow-up, only 1 additional rejection in the q1m group and 2 in the q2m group were recorded, all strongly linked to documented medication nonadherence as was observed during the first year. There were 2 deaths and 2 graft losses in the q2m group, comparable to the 3 deaths and 1 graft loss observed in the q1m group. These results indicate that in low-immunologic-risk patients, every 2-mo dosing is an acceptable and potentially even preferable treatment regimen. Less-frequent q2m belatacept maintenance has potential to facilitate long-term use and better outcomes in kidney transplantation.

Although the initial results of this study at 12-mo follow-up demonstrated equivalent renal function, the higher number of immunologic events in the q2m group raised concerns.^19^ Most immune events were associated with documented noncompliance, suggesting that less-frequent dosing leaves a lower margin for nonadherence. However, this explanation was speculative as limitations of both the phase II study and our noninferiority trial confound our ability to make definitive conclusions regarding the true immunologic risk of q2m dosing.^18,19^ To better approximate this risk, in the most suitable and available methodology, these cohorts were evaluated over 2 additional y of follow-up with a high retention rate. This additional follow-up, although still underpowered to detect subtle differences in immune event rates, provides context to further elucidate the clinical utility of q2m dosing to minimize immunosuppressive burden, enhance convenience, reduce cost, and maintain outcomes. The continued maintenance of renal function as compared with the q1m group observed over this extended period and a narrowing difference in immunologic event rates further support this strategy. The repeated association of immunologic events with medication nonadherence, now observed in both groups with the extended follow-up, supports the viability of q2m dosing in adherent, low-risk patients.

Although the number of immune events on q2m dosing was slightly higher, interestingly, the converse was true for deaths and infectious complications. Four patients in the q1m group experienced clinically significant BK viremia as compared with only 2 in the q2m treatment group. In addition, 1 patient in the q1m group suffered complications from ganciclovir-resistant CMV viremia and, ultimately, passed away. Although overall, there was no difference in cumulative survival from combined death or graft loss between groups‚ it does appear that the q1m group may be more susceptible to death (from infection or otherwise), whereas q2m may be more susceptible to immunologic events associated with nonadherence resulting in graft loss (Table 3; Figure 3). This information could be used in tandem with other immunologic risk factors, for example, eplet disparities,^23^ to inform clinical conversations with patients in determining their optimal immunosuppression. Patients at high risk for nonadherence may do well to remain on monthly dosing, whereas those with a history of infectious complications may benefit from decreased dosing frequency and a reduction in immunosuppressive burden.

Though not statistically significant, the 2 groups did demonstrate different immune event profiles, with a trend toward higher rejection rates in the q2m cohort. When combined with the first year of follow-up, the difference in cumulative survival from acute rejection nearly reaches statistical significance. Although the clinical margin is not dramatic (98.7% 3-y rejection-free survival in q1m versus 92.4% in q2m), these data do suggest that nonadherence may be less tolerated on the q2m dosing regimen. However, the majority of this signal was detected early, with 63% of immune events (5 of 8) in the q2m group observed during the first year of follow-up. The difference in cumulative rejection-free survival from the year 1 time point to the year 3 time point was similar between groups (q1m: −1.3%, q2m: −2.5%, **Table S5, SDC**, http://links.lww.com/TXD/A505). Therefore, although nonadherence may be associated with a higher rate of immune events on a q2m regimen, the resulting events are most likely to occur within the first year during close follow-up. Protocol biopsies were not performed during the transition in this low-immunologic-risk population but could be considered in populations with a higher concern for subclinical events. Unfortunately, without more rigorous documentation of medication adherence between groups, it is difficult to conclusively attribute events to nonadherence. Although clinical coordinators detect and contact patients with missed infusions, oral medications adherence is usually focused on only in the context of clinical concern. Importantly, the 2 adherent subjects (130 and 151) with immune events (1 ACR and 1 DSA, **Table S6, SDC**, http://links.lww.com/TXD/A505) have maintained their baseline renal function without additional immunologic complications 3 y postconversion.

As the field of transplantation continues to strive for improved long-term graft survival, we must consider the comprehensive profile of immunosuppression regimens. Rather than placing undue emphasis on the surrogate outcome of rejection,^24^ immune events should also be viewed in the context of drug and financial toxicity. Due to a paucity of alternatives, belatacept remains the best available maintenance immunosuppressive option in promoting long-term graft function for kidney transplant recipients. In this study, we found that the q2m regimen allowed for improved retention of patients on belatacept therapy, with 98.7% (80 of 81) of patients continuing on a belatacept-based regimen for the duration of the study, compared with 86.5% (71 of 82) in the q1m arm. Belatacept is associated with a different infectious risk profile as well as increased financial burden for many patients.^25^ However, the cost of therapy on a q2m dosing schedule reduces the financial burden of belatacept to approximately that of tacrolimus. At our institution, converting to a q2m regimen translates into $8600 savings per patient per year. Implementing an every-2-mo dosing regimen in patients with low immunological risk allows for optimization of protective immunity for belatacept-treated patients and simultaneously reduces cost and logistical barriers, both of which may contribute to the improved retention on belatacept seen in this group.

Overall, the results of this study support the long-term viability and safety of q2m belatacept dosing in low-immunologic-risk patients. Study limitations include the study design, inclusion of patients over a wide period after transplantation, lack of capture of dose changes in concomitant oral medications, and retrospective nature of the extended 3-y follow-up. Due to feasibility and sample size considerations, the initial randomized trial was not designed with adequate statistical power to detect clinically significant differences in rare immune events, thus hazard ratios could not be precisely estimated for these outcomes. Larger, multicenter clinical trials would be needed to identify underlying differences and to precisely estimate hazard ratios. Additionally, the single-center, selective, and retrospective nature of this analysis limits extrapolation to other patient populations. Nonetheless, q2m subjects exhibited increased similarity to q1m in terms of immunologic outcomes over the long-term, with improved retention on belatacept and fewer deaths from infectious complications. Implementation of a q2m dosing regimen may allow for increased use of costimulation blockade-based immunosuppression by reducing financial, logistic, and infectious barriers to adoption of belatacept therapy. Further characterization of outcomes by a multicenter approach may lead to improved understanding of the efficacy of q2m dosing in a broader patient population. Continued study is warranted to better define the patient populations likely to benefit from reduced belatacept dosing frequency.

## Supplementary Material


