# Supplementary material for: Three-year Outcomes After Conversion From Monthly to Every 2-month Belatacept Maintenance Therapy in Kidney Transplant Recipients: Results From a Randomized Controlled Trial
Source: Transplant Direct. 2023 Feb 8;9(3):e1449. doi: 10.1097/TXD.0000000000001449 (PMC9977481; doi:10.1097/TXD.0000000000001449)

Tables S1. Means and 95% confidence intervals for eGFR by treatment group and months on study (0-3 years) using unadjusted values. 163 subjects with 3771 eGFR measurements.

	Q1: Monthly intravenous infusions of Belatacept				Q2: Every 2-month intravenous infusions of Belatacept			
**Month**	**N**	**Mean eGFR**	**95% Lower CI**	**95% Upper CI**	**N**	**Mean eGFR**	**95% Lower CI**	**95% Upper CI**
0	82	72.5	68.7	76.3	81	69.2	65.7	72.8
1	77	72.7	69.0	76.5	79	69.1	65.3	72.9
2	79	71.9	67.9	75.9	79	70.8	66.9	74.7
3	77	71.4	67.3	75.4	71	70.8	66.8	74.8
4	77	73.2	69.0	77.3	78	71.1	67.2	75.0
5	74	73.2	69.1	77.2	75	70.4	66.6	74.2
6	78	73.5	69.4	77.5	80	70.2	66.2	74.2
7	73	72.1	67.7	76.4	77	71.6	67.7	75.6
8	74	72.1	67.9	76.2	80	70.6	66.4	74.8
9	74	72.7	68.1	77.2	74	70.7	66.7	74.6
10	72	73.5	68.9	78.0	79	69.7	65.7	73.7
11	69	74.6	70.4	78.9	71	68.3	64.2	72.3
12	76	72.8	68.7	76.9	80	69.2	65.4	73.0
13-15	71	71.4	67.1	75.6	77	68.8	64.8	72.9
16-18	63	71.3	66.8	75.8	78	70.7	66.4	75.0
19-21	70	73.2	68.8	77.6	73	70.2	66.2	74.3
22-24	71	73.5	69.2	77.8	75	69.2	64.8	73.5
25-27	70	74.3	69.8	78.7	70	69.6	65.0	74.3
28-30	62	73.4	69.2	77.7	74	68.5	63.8	73.2
31-33	64	73.8	69.5	78.1	75	68.4	63.6	73.2
34-36	68	74.9	70.2	79.5	70	69.8	65.1	74.4
Overall	82	**72.9**	69.1	76.8	81	**69.9**	66.2	73.5

Time-averaged eGFR mean by treatment group and Mean Difference

Group	**N**	**Mean eGFR**	**95% Lower CI**	**95% Upper CI**
Q1m	82	72.9	69.1	76.8
Q2m	81	69.9	66.2	73.5

Mean Difference		3.0	-2.3	8.4

Tables S2. Baseline-adjusted means and 95% confidence intervals for eGFR by treatment group and months on study (0-3 years). N=163 with 2954 GFR measurements.

	**Q1: Monthly intravenous infusions of Belatacept**				**Q2: Every 2-month intravenous infusions of Belatacept**			
**Month**	**N**	**Mean eGFR**	**95% Lower CI**	**95% Upper CI**	**N**	**Mean eGFR**	**95% Lower CI**	**95% Upper CI**
1	77	71.7	69.8	73.5	79	70.3	68.4	72.3
2	79	70.8	69.0	72.6	79	72.2	70.1	74.4
3	77	71.0	68.9	73.2	71	71.9	69.9	73.8
4	77	72.6	70.4	74.9	78	72.2	69.8	74.5
5	74	72.6	70.2	75.0	75	71.9	69.8	74.0
6	78	72.3	70.1	74.6	80	71.3	69.2	73.4
7	73	70.5	67.9	73.1	77	73.0	70.7	75.3
8	74	70.3	68.1	72.6	80	71.8	69.0	74.7
9	74	71.7	69.3	74.0	74	72.3	70.2	74.4
10	72	72.5	69.9	75.2	79	71.7	69.4	74.0
11	69	73.5	71.2	75.8	71	70.3	67.8	72.9
12	76	71.5	69.2	73.7	80	71.3	68.9	73.6
13-15	71	69.8	67.3	72.4	77	70.3	67.7	72.9
16-18	63	70.1	67.1	73.0	78	72.4	69.8	75.1
19-21	70	71.9	68.8	74.9	73	72.0	69.2	74.8
22-24	71	72.4	69.7	75.1	75	71.2	67.8	74.6
25-27	70	72.6	69.9	75.3	70	71.7	67.6	75.8
28-30	62	72.1	69.3	74.9	74	70.7	67.2	74.1
31-33	64	71.7	68.8	74.5	75	70.3	66.5	74.1
**34-36**	**68**	**72.9**	**69.9**	**76.0**	**70**	**71.6**	**68.2**	**75.0**
Overall	82	**71.7**	70.0	73.5	81	**71.5**	69.5	73.5

Baseline-adjusted Time-averaged eGFR means by treatment group

**Treatment**	**N**	**Mean eGFR**	**95% Lower CI**	**95% Upper CI**
Q1m	82	71.7	70.0	73.5
Q2m	81	71.5	69.5	73.5

**Mean Treatment Difference**<br>**(Q1m-Q2m)**		0.2	-2.5	2.9

P (time)= 0.09, P(group)=0.88, P(interaction)=0.16, and P (baseline GFR)= <.0001

Tables S3. Sensitivity Analysis 1. Baseline-adjusted means and 95% confidence intervals for eGFR by treatment group and months on study (0-3 years) for patients conditional of patient and graft survival. N=153 survivors without graft failure (2814 GFR measurements).

	Q1: Monthly intravenous infusions of Belatacept				Q2: Every 2-month intravenous infusions of Belatacept			
**Month**	**N**	**Mean eGFR**	**95% Lower CI**	**95% Upper CI**	**N**	**Mean eGFR**	**95% Lower CI**	**95% Upper CI**
1	71	71.6	69.7	73.5	75	71.0	69.1	72.9
2	73	70.9	69.0	72.7	75	72.7	70.6	74.8
3	71	70.5	68.5	72.6	67	72.3	70.3	74.2
4	71	72.6	70.5	74.7	74	72.7	70.4	74.9
5	69	72.0	69.6	74.3	71	72.0	69.9	74.1
6	74	72.9	70.8	75.1	76	71.9	70.0	73.9
7	69	72.0	69.9	74.2	73	73.1	70.8	75.3
8	70	71.8	69.9	73.6	76	72.7	70.6	74.9
9	71	72.7	70.5	74.8	70	72.3	70.5	74.0
10	70	73.3	71.1	75.6	75	72.5	70.4	74.5
11	66	74.4	72.6	76.3	68	71.1	69.1	73.1
12	73	72.5	70.5	74.5	76	71.9	70.1	73.7
13-15	67	71.5	69.7	73.4	73	71.6	69.4	73.8
16-18	61	70.9	68.3	73.5	74	73.4	71.4	75.4
19-21	67	72.7	70.2	75.1	69	72.7	70.5	74.9
22-24	68	73.7	71.7	75.7	71	72.6	70.4	74.8
25-27	69	73.6	71.0	76.2	66	73.8	71.3	76.3
28-30	61	73.3	70.6	76.0	71	72.6	70.2	75.0
31-33	63	73.3	70.7	76.0	73	72.5	69.9	75.0
34-36	68	74.4	71.6	77.1	69	72.9	70.3	75.5
Overall	76	72.5	71.0	74.0	77	72.4	70.9	73.9

Time-averaged eGFR baseline-adjusted means by treatment group

Group	**N**	**Mean eGFR**	**95% Lower CI**	**95% Upper CI**
Q1m	76	72.5	71.0	74.0
Q2m	77	72.4	70.9	73.9

**Mean Treatment Difference (Q1m-Q2m)**		0.1	-2.0	2.2

Tables S4. Sensitivity analysis 2. Baseline-adjusted means and 95% confidence intervals for eGFR by treatment group and months on study (0-3 years). eGFR imputed as zero after graft loss or death.

	Q1m: Monthly intravenous infusions of Belatacept				Q2m: Every 2-month intravenous infusions of Belatacept			
**Month**	**N**	**Mean eGFR**	**95% Lower CI**	**95% Upper CI**	**N**	**Mean eGFR**	**95% Lower CI**	**95% Upper CI**
1	77	71.4	69.4	73.4	79	70.4	68.3	72.5
2	79	70.9	69.1	72.8	79	72.3	70.0	74.6
3	77	70.7	68.5	72.9	71	72.2	70.1	74.2
4	77	72.7	70.3	75.0	78	72.0	69.7	74.4
5	75	72.1	69.1	75.0	75	71.8	69.7	73.9
6	80	71.2	68.1	74.3	80	72.1	69.5	74.6
7	75	69.7	66.4	73.1	77	73.3	70.6	76.1
8	76	69.3	65.6	73.0	80	71.2	68.4	74.1
9	77	70.3	66.5	74.2	74	71.7	69.5	73.9
10	76	70.1	66.4	73.9	79	71.1	68.5	73.8
11	72	71.9	68.6	75.2	72	69.0	65.3	72.8
12	79	69.4	66.1	72.6	80	70.6	68.2	73.0
13-15	73	68.5	65.5	71.4	77	71.3	68.5	74.1
16-18	67	66.7	62.5	70.9	78	72.9	70.2	75.6
19-21	73	68.4	63.9	72.8	73	72.3	69.4	75.1
22-24	74	69.4	65.6	73.2	75	71.2	68.4	74.1
25-27	75	68.1	63.1	73.1	70	72.0	68.5	75.5
28-30	67	68.4	63.8	73.1	75	68.4	64.1	72.6
31-33	69	68.3	63.3	73.3	77	68.0	63.6	72.3
34-36	74	68.5	63.7	73.2	73	68.7	64.3	73.1
Overall	82	69.8	67.2	72.4	81	71.1	69.1	73.1

Time-averaged eGFR baseline-adjusted means by treatment group

Group	N	Mean eGFR	95% Lower CI	95% Upper CI
Q1m	82	69.8	67.2	72.4
Q2m	81	71.1	69.1	73.1
**Mean Treatment Difference (Q1m-Q2m)**		-1.3	-4.6	2.0

Table S5. Cumulative survival rates by cohort at the 1, 2, and 3 year time points.

Time Point	1 year	2 year		3 year
**Rejection-Free Survival**				
Q1	100%[100, 100]	98.7%[96.2, 100]		98.7%[96.2, 100]
Q2	94.9%[90.2, 99.9]	93.7%[88.5, 99.2]		92.4%[86.7, 98.4]
**DSA-Free Survival**				
Q1	100%[100, 100]	98.7%[96.2, 100]		98.7%[96.2, 100]
Q2	96.2%[92.1, 100]			
**Survival without Immune Event**	98.7%[96.2, 100]		94.9%[90.2, 99.9]	9
Q1	93.7%[88.5, 99.2]			89.9%[83.4,
Q2		92.4%[86.7, 98.4]	97.4%[93.9, 100]	996.8]
**Survival without Death or Graft Loss**				
Q1		97.6[94.3, 100]	95.1%[90.6, 99.9]	92.6%[87.1, 98.5]
Q2		100%[100, 100]	100%[100, 100]	95.0%[90.4, 99.9]

Table S6. Follow-up through years 2 and 3 of patients who experienced immunologic events during the first year of the study.

SUBJECT	TIME	GROUP	PATHOLOGY	DSA SPECIFICITY (MAX MFI)	ACTION/COMMENT	FOLLOWUP (M)
**009**	6, 10	Q1M	Borderline x 2	No DSA	Oral steroid pulse x 2	Died of unknown causes at month 24
**078**	6	Q2M	1A ACR	No DSA	IV steroids. Converted to q1m.	Stable renal function
**098**	8, 15	Q2M	1B ACR/AMR, borderline	DQ (3600)	IV steroids, thymoglobulin, IVIG/plasmapheresis. Converted to q1m.	Continued NC, and progression to graft failure
**130**	4	Q2M	1A ACR	No DSA	Converted to q1m	No further events
**135**	4, 13	Q2M	1B ACR, AMR, borderline	DQ (2635)	Thymoglobulin, plasmapheresis/IVIG. Converted to q1m. Received oral steroid pulse for borderline rejection.	Stable renal function
**151**	6	Q2M	Bland biopsy	DQ (10633)	Converted to q1m	No further events

Abbreviations: NC = noncompliance, eGFR = estimated glomerular filtration rate, Q1M = every month dosing, Q2M = every 2-month dosing of belatacept, ACR = acute cellular rejection, AMR = antibody mediated rejection, MMF = mycophenolate mofetil.

Figure S1. Cumulative survival from any adverse event (DSA, rejection, death, or graft loss).

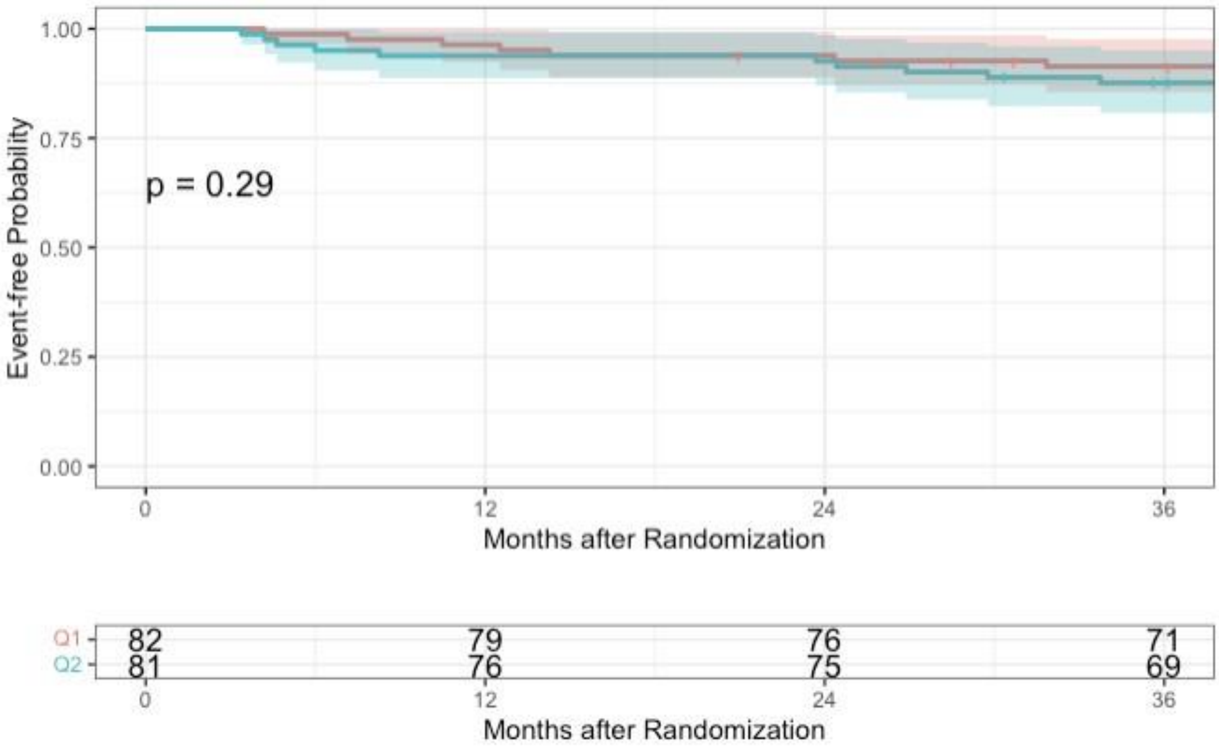

Supplement: Supplementary file 1 [file txd-9-e1449-s001.pdf]
